# Incarceration, Social Support Networks, and Health among Black Sexual Minority Men and Transgender Women: Evidence from the HPTN 061 Study

**DOI:** 10.3390/ijerph191912064

**Published:** 2022-09-23

**Authors:** Joy D. Scheidell, Farzana Kapadia, Rodman E. Turpin, Medha Mazumdar, Typhanye V. Dyer, Jonathan Feelemyer, Charles M. Cleland, Russell Brewer, Sharon D. Parker, Natalia M. Irvine, Molly Remch, Kenneth H. Mayer, Maria R. Khan

**Affiliations:** 1 Department of Population Health, New York University Grossman School of Medicine, New York, NY 10016, USA; 2 School of Global Public Health, New York University, New York, NY 10012, USA; 3 School of Public Health, Epidemiology and Biostatistics, University of Maryland at College Park, College Park, MD 20742, USA or; 4 Department of Global and Community Health, College of Health and Human Services, George Mason University, Fairfax, VA 22030, USA; 5 Department of Medicine, University of Chicago, Chicago, IL 60637, USA; 6 Department of Social Work, North Carolina Agricultural and Technical State University, Greensboro, NC 27411, USA; 7 Department of Epidemiology, UNC Gillings School of Global Public Health, University of North Carolina at Chapel Hill, Chapel Hill, NC 27599, USA; 8 Department of Global Health and Population, Harvard T.H. Chan School of Public Health, Harvard University, Boston, MA 02115, USA; 9 The Fenway Institute, Fenway Health, Boston, MA 02215, USA

**Keywords:** incarceration, social networks, sexual minorities

## Abstract

Support from social networks buffers against negative effects of stress but is disrupted by incarceration. Few studies examine incarceration, social support networks, and health among Black sexual minority men (BSMM) and Black transgender women (BTW). We conducted a secondary analysis using HIV Prevention Trials Network 061 (HPTN 061), a sample of BSMM/BTW recruited from six US cities. We measured associations between recent incarceration reported at six months follow-up and social support networks at twelve months follow-up, and cross-sectional associations between support networks and twelve-month health outcomes (e.g., sexual partnerships, substance use, healthcare access and depressive symptoms). Among the analytic sample (N = 1169), recent incarceration was associated with small medical support networks (adjusted risk ratio [aRR] 1.16, 95% CI 1.01, 1.34) and small financial support networks (aRR 1.18, 95% CI 1.04, 1.35). Support networks were associated with multiple partnerships (adjusted prevalence ratio [aPR] 0.77, 95% CI 0.65, 0.90), unhealthy alcohol use (aPR 1.20, 95% CI 0.96, 1.51), and depressive symptoms (aPR 1.16, 95% CI 0.99, 1.36). Incarceration adversely impacts social support networks of BSMM/BTW, and support networks were associated with a range of important health outcomes.

## 1. Introduction

Social networks provide support across a range of domains, including emotional, financial, and medical, and are vital for a person’s health. The stress-buffering hypothesis of social support posits that the size and quality of one’s social networks can mitigate the negative effects of stress on health [1,2]. The ameliorating effects of support from one’s networks may be especially important for Black sexual minority men (BSMM) and Black transgender women (BTW), considering that minority stress theory suggests overall stress combined with stress experienced related to one’s identities in minority groups may increase vulnerability to discrimination and subsequent adverse health [3]. In the United States (US), there are no federal laws that prohibit discrimination in public accommodations based on sexual orientation or gender identity [4], and although lesbian, gay, bisexual, and transgender (LGBT) acceptance has increased in the past two decades, the US lags behind similar countries such as Canada [5]. Among sexual minorities, aspects of social networks, such as the size and the roles of relationships within them, protect against negative effects of stress and discrimination on health, depression, and life satisfaction [6]. Among BSMM and BTW, most research focuses on HIV risk and associated factors such as depression, violence, and substance use, and shows the protective role of social networks [7,8,9,10,11,12].

Disrupting social networks increases the spread of disease and negatively affects health [13,14]. Mass incarceration in the US is a population-level driver of network disruption, and is likely a mechanism by which incarceration increases risk of disease and other negative health outcomes [15]. Despite declines in incarceration rates since their height in 2009 [16], rates of incarceration in the US remain high [17]. Importantly, structural racism inherent in the US criminal justice system has led to Black men experiencing incarceration at rates at least five times higher than their white counterparts [18]. Racial/ethnic inequity in incarceration is exacerbated among sexual and gender minority persons, with up to two-thirds of BSMM and 40% of BTW experiencing incarceration during their lifetime, and substantial proportions incarcerated annually [19,20,21,22,23].

Incarceration separates people from their social networks and often leads to network instability upon release. During re-entry, people report inconsistent contact with network members and that their post-release network consists of different people [24,25]. Post-release network instability may drive those who were formerly incarcerated into networks with higher risk of drug use or to replace stable sexual network members with new, additional, and/or concurrent partners [26,27,28]. While this is likely true regardless of race, incarceration-related network disruption and instability may be particularly salient for BSMM and BTW. The excessively high rates of incarceration among Black people disproportionately impacts their networks [29], and their social networks may be more sensitive to turnover and instability [30]. Among Black sexual and gender minority persons, non-family social networks have often been constructed as alternative familial and kinship networks in response to exclusion from biological or other heteronormative networks [31,32]. Compared to heterosexual individuals, sexual minority people receive fewer dimensions of social support from their networks [33], and the sexual networks of young BSMM who have been incarcerated are more likely to contain members who have also been incarcerated [34]. Therefore, lacking support and network turnover may be further heightened for BSMM and BTW during the re-entry period.

There has been little examination of incarceration, social support networks, and health among BSMM and BTW. In the current study, we examined the relationship between incarceration and subsequent social support network characteristics, and explored whether social support networks were associated with health outcomes in a sample of BSMM and BTW in six US cities.

## 2. Materials and Methods

### 2.1. Sample and Study Design

The HIV Prevention Trials Network (HPTN) 061 cohort has been described in detail previously [35]. In brief, HPTN 061 was a longitudinal cohort study that enrolled 1553 participants in 2009–2010 from Atlanta, New York City, Washington DC, Los Angeles, San Francisco and Boston; who were at least 18 years old; self-identified as male or being assigned male at birth; self-identified as Black, African American, Caribbean Black, or multiethnic Black; and reported at least one condomless anal intercourse event with a male partner in the past six months. At study visits conducted at baseline, 6-, and 12-month follow up, participants completed surveys using audio computer assisted self-interviewing technology that measured topics including criminal justice involvement, sexual behaviors, substance use, and mental health. Participants completed social network inventories at baseline and the 12-month visit. Biological specimens were collected for STI and HIV testing (i.e., syphilis and HIV assessed via blood; gonorrhea and chlamydia assessed via urine/rectal swab).

### 2.2. Measures

Incarceration. At the six-month follow-up visit, participants reported the number of times that they had spent one or more nights in jail or prison in the past six months; those who reported they had spent at least one night incarcerated were defined as having experienced recent incarceration.

Social Support Networks. A social network inventory was completed by the participants to assess perceived social support [36]. Participants were asked to name up to five persons they could rely on for the following forms of support: (1) medical support, defined as “Is there anybody who would go to a medical appointment with you?”; (2) financial support, defined as “Is there anybody you know who you would ask to lend you $100 or more if you needed it?”; (3) social support, defined as “Is there anybody that you get together with, spend time talking, relaxing or just hanging out with?”; and (4) emotional support, defined as “If you wanted to talk to someone about things that are very personal and private is there anybody you could talk to?” We created dichotomous indicators for each type of support, in which each item was dichotomized to capture “small” support networks, defined as ≤1 member in the network providing that form of support (versus ≥2 members).

Using the above measures, we created three additional support measures. The first dichotomous variable measured having a consistent person in their network providing all four forms of social support, in which the same individual provided each form of support. The second variable measured whether participants reported that they had ≥1 network member providing each form of support for all four support domains; this did not need to be the same person. The third variable identified those who had ≤1 person in network providing each form of support for all four support domains (i.e., categorized as having “small” support networks as defined above for all forms of support).

Health Outcomes. We measured the following outcomes, which were self-reported on the 12-month follow-up survey: multiple sexual partnerships, defined as reporting ≥3 partners (i.e., sample median) in the past six months; hard drug use, defined as any use of heroin, crack/cocaine, methamphetamine, prescription misuse, or other drugs in the past six months; unhealthy alcohol use, defined as having an AUDIT (Alcohol Use Disorders Identification Test) score ≥ 8; emergency department (ED) use, defined as having any care provided to them at an emergency room or urgent care facility in past six months; and depressive symptoms, based on Centers for Epidemiologic Studies–Depression scale score ≥ 16 [37].

Covariates. Baseline covariates included self-reported measures of: recruitment city; age; gender identity; unstable housing; high school education or less; insufficient income in the past six months; hard drug use in the past six months; weekly marijuana use; current healthcare coverage; lifetime incarceration; unhealthy alcohol use; depressive symptoms; physical and/or threatened violence due to race and/or sexuality; perceived racism and homophobia [38]; internalized homophobia [39]; social support scale score; sexual behavior in the past six months (i.e., sex with female partners; transactional sex; multiple partnerships; concurrent partnership defined as partners in addition to their primary partner); HIV testing; and currently cohabiting with a primary partner. Biologically ascertained covariates included baseline HIV status and any STI.

### 2.3. Analyses

Our analytic sample included participants who returned for the six-month visit who had data on recent incarceration (N = 1169). Scales with missing values were replaced with the mean value of the remaining items if fewer than 20% of items were missing; when more than 20% scale items were missing, the score was coded as missing. Approximately 77% of participants in the analytic sample were missing data on at least one covariate, and multiple imputation was used to reduce bias and increase power in the analyses by imputing data 77 times using predictive mean matching in the “mice” package.

The propensity (i.e., predicted probability) of recent incarceration was calculated using logistic regression with the Ridge penalty conditional on the baseline covariates above, including sociodemographic characteristics, sexual risk behavior, substance use, and experienced and internalized racism and homophobia. Propensity scores were used to estimate inverse probability of treatment weights (IPTW), which were stabilized using the probability of the observed exposure. Models were conducted for each of the 77 imputed datasets.

We examined baseline factors associated with having ≤1 person in the network providing each form of support and having a consistent person in their network by calculating the frequency and prevalence of each covariate by these measures. We used unweighted and weighted modified Poisson regressions with robust standard errors to assess the associations between recent incarceration measured at six months and the social support network variables measured at 12 months, and estimated risk ratios and 95% confidence intervals (CIs) within each of the imputed datasets by extracting parameter estimates and variances from each model and pooling following Rubin’s rules. The pooled results are presented.

We used modified Poisson regressions with robust standard errors to estimate prevalence ratios and 95% confidence intervals for associations between social network support and health outcomes at 12 months. In multivariate models, we adjusted for baseline age, education, household income, housing status, city of recruitment, reporting a place to go for medical care when sick, having seen health care provider in the past six months and the corresponding outcome reported at baseline (e.g., adjusting for baseline multiple partnership for the multiple partnership outcome). R version 4.0.5 (R Core Team, Vienna, Austria) was used for analysis [40].

## 3. Results

In the sample of 1169 (BSMM n = 1118; BTW n = 49), approximately half of participants reported ≤1 member in their network provided medical or financial support (i.e., small support networks), 26% reported small social support networks, and 40% reported small emotional support networks. For composite network support indicators, 46% of participants reported they had a consistent person in their network (i.e., someone who provided all forms of support), 65% reported ≥1 person providing support for each domain, and 16% reported ≤1 person in their network provided support for each domain (i.e., all support networks were defined as small). Among those with a consistent person in their network, those who had been incarcerated, versus those without incarceration, were more likely to report this person was a sexual partner (37.3% versus 25.1%; *p*-value 0.04) and less likely to report this person was a friend (45.8% versus 63.6%; *p*-value 0.008). Approximately one-third reported this was a family member, which did not vary by incarceration.

Participants who reported having less than a high school education, multiple sexual partnerships, and engaging in sex trade had higher odds of small support networks (Table 1). However, individuals who reported having sufficient income, receiving HIV testing, having healthcare coverage and a place to go for medical care, and having sex with men and women had lower odds of having small support networks. Increasing age and having less than a high school-level education was associated with lower odds of a consistent person in the network. Importantly, people with lifetime incarceration history, unstable housing, and sex trade engagement had lower odds of consistency in network membership; conversely, those who reported sufficient income and having sex with both men and women had higher odds.

Those who were recently incarcerated were more likely to report small support networks compared to those who were not recently incarcerated (Table 2), and were less likely to have a consistent network member, have multiple persons in their networks, and have one or fewer network members providing all forms of support. In the adjusted models applying the IPTW, effect estimates were relatively similar to unadjusted models and showed that recent incarceration was associated with a small medical support network (adjusted risk ratio [aRR] 1.16, 95% CI 1.01, 1.34) and a small financial support network (aRR 1.18, 95% CI 1.04, 1.35) but was not associated with other indicators of network support.

In the cross-sectional analyses examining the relationship between social support networks and health outcomes reported at 12 months follow-up in the sample (Table 3), the prevalence of multiple partnerships was approximately 20% lower for those reporting small social support networks, consistent network member, and ≤1 person in the network providing each form of support. Having small medical support network (aPR 1.20, 95% CI 0.96, 1.51), small financial support network (aPR 1.15, 95% CI 0.94, 1.41), small emotional support network (aPR 1.16, 95% CI 0.94, 1.42) were associated with unhealthy alcohol use. Small emotional support network was associated with visiting the ED (aPR 1.22, 95% CI 0.99, 1.50). Small financial support networks (aPR 1.12, 95% CI 0.96, 1.30), small social support networks (aPR 1.16, 95% CI 0.99, 1.36), small emotional support networks (APR 1.12, 95% CI 0.95, 1.33), having one person or fewer in the network providing each form of support (aPR 1.22, 95% CI 1.02, 1.46) were associated with depressive symptoms, as was reporting consistent network support (aPR 0.85, 95% CI 0.73, 0.99).

## 4. Discussion

Among this sample of BSMM and BTW, recent incarceration was associated with having few network members providing medical and financial support after release. These findings suggest the need to mitigate the negative impact of incarceration on the social support networks of BSMM and BTW, potentially by increasing alternatives to incarceration along with programming to maintain support networks during incarceration. We observed some evidence that medical and financial support networks were protective against depression, alcohol use, and ED visitation, though results were not conclusive. These findings highlight the potential importance of social support networks for a range of health outcomes among BSMM and BTW and the need for additional research on the link between incarceration-related network disruption and health in this group.

Prior studies on incarceration and social networks have focused on networks as risk factors for incarceration [41], formation of networks within correctional facilities [42,43,44], and incarceration-related disruption of opposite-sex partnerships [27,28]. Our study is among the first to demonstrate deleterious effects of incarceration on social networks among BSMM and BTW. We found that those who had been incarcerated appeared less likely to have a consistent person in their network, and among those that did, participants who had been incarcerated were more likely to report that this person was a sexual partner. Among Black cisgender men, incarceration was associated with disrupting committed sexual partnerships with women [27,28]. Although we do not know if the person who was consistent across networks post release was the same as prior to incarceration, our results demonstrated the importance of having a consistent network member providing support and underscore the importance of sexual partners as a source of support for those who have been incarcerated, and the importance of non-familial network members overall for BSMM and BTW.

Our results indicate that incarceration may also affect network size, with people who were recently incarcerated reporting small medical and financial support networks. The sparse literature in this area is mixed. Some studies found that network size is reduced during the post-release period [42], whereas others have reported no difference pre- and post-incarceration [45]. However, these studies measured overall network size or networks defined by risk behavior (i.e., substance use) rather than forms of support from the networks. We did not observe an association between incarceration and the size of one’s emotional or social support networks, which may suggest the intimacy with network members who provide medical and financial support is more vulnerable to disruption from incarceration compared to the emotional and social support that may be provided by network members with more superficial relationships.

The importance of social support networks for health is well documented, but has focused mostly on STI/HIV risk among BSMM and BTW [7,8,9,10,11,12,46]. Our results extend to other important health outcomes, including substance use and health care utilization. We did not observe statistically significant relationships between social support networks and drug use, which is counter to hypotheses that people who are incarcerated may become enmeshed in drug use networks [41,45]. Social support networks were associated with unhealthy alcohol use, with small network size associated with higher prevalence, while having a consistent person network member was associated with lower prevalence. This supports prior evidence that disruption of partnerships during incarceration is linked to post-release binge drinking [47]. One’s networks’ substance use and incarceration explains how incarceration influences individual’s post-release substance use [34,45], but in this study we lack information on network members’ substance use. We also do not have information on the substance use norms within one’s networks, which are powerful drivers of an individual’s risk behavior and targets for network-based interventions [8,48,49].

Additional limitations must be noted. First, while we controlled for baseline measures of support networks, we lack data regarding the stability of networks before and after incarceration, and prior research has found that the number of people in one’s network may be stable after incarceration but that there is high turnover of people within the network [25]. We cannot measure other sources of social support that may be important for health during re-entry such as case managers [50,51]. There was also limited data on non-sexual network members and homophily (i.e., similarity) related to sexual minority status, which may confer specific protection for BSMM and BTW [6]. HPTN 061 was not focused on recruiting BTW and the small number enrolled does not allow for adequate statistical power to examine differences in the associations among BSMM versus BTW [9]. Analyses of support networks and health were cross-sectional, and we cannot ascertain temporality. For example, someone experiencing depressive symptoms may withdraw from their social networks. Our measures were self reported and subject to social desirability and recall bias. We included numerous baseline covariates that may effect recent incarceration, social support networks, and health outcomes in our IPTW models to account for their potential confounding effects. However, the relationships among the covariates, such as experiences and internalization of homophobia, likely intersect and are complex, warranting future research. Finally, our sample may not be generalizable to other BSMM and BTW.

## 5. Conclusions

This study is among the first to show that incarceration is associated with the size and composition of social support networks of BSMM and BTW, and that these support network characteristics are associated with a range of important health outcomes. Future research is needed to better characterize changes in social support networks pre- and post-incarceration and to develop interventions to bolster support networks during incarceration while also limiting exposure to incarceration among BSMM and BTW. Reducing the disproportionate levels of incarceration among BSMM and BTW is crucial to prevent subsequent adverse consequences on social support and health. Policies to address racism and homophobia in the criminal legal system to prevent unequal targeting of racial and/or gender minority people are needed, as is programming to support social support networks during incarceration and re-entry.

## Figures and Tables

**Table 1:** Baseline Characteristics of HPTN 061 Sample and Associations with Network Size and Types at 12 Months (N = 1169).

Characteristic at Baseline	Overall N (%) N = 1169	N (%) with One Person or Fewer in Network Providing Each Form of Support N = 192	OR * (95% CI) for Association between Characteristic and One Person or Fewer in Network Providing Each Form of Support	*p*-Value for Association between Characteristic and One Person or Fewer in Network Providing Each Form of Support	N (%) with Consistent Network Support N = 542	OR * (95% CI) for Association between Characteristic and Consistent Network Support	*p*-Value for Association between Characteristic and Consistent Network Support
**Age**							
Mean (SD)	37.7 (11.8)	38.40 (12.24)	1.01 [0.99, 1.02]	0.402	37.1 (12.1)	0.98 [0.97, 0.99]	0.001
**Education**							
High School or More	568 (48.6)	74 (13.0)	Referent		307 (54.0)	Referent	
Less than High School	601 (51.4)	118 (19.6)	1.84 [1.34, 2.54]	<0.001	235 (39.1)	0.61 [0.48, 0.78]	<0.001
**Transgender**							
No	1118 (95.6)	182 (16.3)	Referent		522 (46.7)	Referent	
Yes	49 (4.2)	10 (20.4)	1.32 [0.61, 2.62]	0.455	20 (40.8)	0.76 [0.41, 1.39]	0.371
**City**							
Atlanta	207 (17.7)	52 (25.1)	Referent		68 (32.9)	Referent	
Boston	173 (14.8)	31 (17.9)	0.71 [0.42, 1.18]	0.192	64 (37.0)	1.41 [0.90, 2.21]	0.131
Los Angeles	207 (17.7)	39 (18.8)	0.68 [0.42, 1.10]	0.117	109 (52.7)	2.50 [1.65, 3.83]	<0.001
New York City	256 (21.9)	42 (16.4)	0.53 [0.33, 0.85]	0.008	116 (45.3)	1.58 [1.07, 2.35]	0.022
San Francisco	149 (12.7)	13 (8.7)	0.27 [0.13, 0.50]	<0.001	86 (57.7)	2.94 [1.86, 4.69]	<0.001
Washington DC	177 (15.1)	15 (8.5)	0.25 [0.13, 0.45]	<0.001	99 (55.9)	2.51 [1.64, 3.89]	<0.001
**Incarcerated Ever**							
No	465 (39.8)	72 (15.5)	Referent		238 (51.2)	Referent	
Yes	686 (58.7)	117 (17.1)	1.14 [0.83, 1.59]	0.420	299 (43.6)	0.73 [0.57, 0.94]	0.013
**Experienced Violence**							
No	284 (24.3)	37 (13.0)	Referent		135 (47.5)	Referent	
Yes	866 (74.1)	152 (17.6)	1.40 [0.95, 2.09]	0.094	400 (36.8)	0.90 [0.68, 1.20]	0.474
**Insufficient Income**							
Yes	655 (56.0)	132 (20.2)	Referent		262 (40.0)	Referent	
No	513 (43.9)	60 (11.7)	0.49 [0.35, 0.68]	<0.001	280 (54.6)	1.76 [1.37, 2.25]	<0.001
**Unstable Housing**							
No	1055 (90.2)	171 (16.2)	Referent		507 (48.1)	Referent	
Yes	113 (9.7)	21 (18.6)	1.27 [0.74, 2.08]	0.361	35 (31.0)	0.50 [0.32, 0.76]	<0.001
**Ever tested for HIV**							
No	140 (12.0)	31 (22.1%)	Referent		59 (42.1)	Referent	
Yes	1028 (87.9)	161 (15.7%)	0.62 [0.40, 0.97]	0.031	483 (47.0)	1.16 [0.79, 1.70]	0.446
**Health Care Coverage**							
No	456 (39.0)	90 (19.7)	Referent		203 (44.5)	Referent	
Yes	712 (60.9)	102 (14.3)	0.66 [0.48, 0.91]	0.011	339 (47.6)	1.12 [0.87, 1.44]	0.363
**Place to Go for Medical Care when Sick**							
No	247 (21.1)	54 (21.9)	Referent		109 (44.1)	Referent	
Yes	922 (78.9)	138 (15.0)	0.61 [0.43, 0.88]	0.008	433 (47.0)	1.12 [0.83, 1.51]	0.465
**Seen a Medical Provider in Past 6 Months**							
No	462 (39.5)	87 (18.8)	Referent		226 (48.9)	Referent	
Yes	707 (60.5)	105 (14.9)	0.73 [0.53, 1.00]	0.052	316 (44.7)	0.80 [0.62, 1.02]	0.076
**Sexual Partnership Types**							
Men Only	511 (43.7)	109 (21.3)	Referent	<0.001	187 (36.6)	Referent	
Men and Women	657 (56.2)	83 (12.6)	0.48 [0.35, 0.65]		355 (54.0)	1.88 [1.47, 2.42]	<0.001
**Multiple Partnership**							
No	673 (57.6)	97 (14.4)	Referent		323 (48.0)	Referent	
Yes	494 (42.3)	95 (19.2)	1.43 [1.04, 1.96]	0.025	219 (44.3)	0.85 [0.67, 1.09]	0.205
**Concurrency**							
No	882 (75.4)	141 (16.0)	Referent		399 (45.2)	Referent	
Yes	287 (24.6)	51 (17.8)	1.07 [0.74, 1.52]	0.709	143 (49.8)	1.10 [0.83, 1.46]	0.500
**Lives with Primary Partner**							
No	975 (83.4)	158 (16.2)	Referent		446 (45.7)	Referent	
Yes	177 (15.1)	31 (17.5)	1.04 [0.67, 1.58]	0.857	90 (50.8)	1.14 [0.81, 1.59]	0.452
**Any STI**							
No	1010 (86.4)	171 (16.9)	Referent		460 (45.5)	Referent	
Yes	138 (11.8)	19 (13.8)	0.74 [0.43 1.21]	0.252	72 (52.2)	1.22 [0.84, 1.78]	0.293
**Sex Trade**							
No	872 (74.6)	126 (14.4)	Referent		422 (48.4)	Referent	
Yes	297 (25.4)	66 (22.2)	1.68 [1.20, 2.35]	0.002	120 (40.4)	0.68 [0.51, 0.90]	0.006
**HIV Status at Baseline**							
HIV+ Acute	3 (0.3)	1 (33.3)	Referent		1 (33.3)	Referent	
Negative	935 (80.0)	145 (15.5)	0.43 [0.04, 9.24]	0.490	426 (45.6)	2.15 [0.20, 46.29]	0.533
Positive	214 (18.3)	45 (21.0)	0.58 [0.05, 12.58]	0.656	105 (49.1)	2.19 [0.21, 47.51]	0.525
Unknown	16 (1.4)	1 (6.2)	0.14 [0.00, 4.56]	0.225	9 (56.2)	3.00 [0.24, 73.58]	0.410
**Hard Drug Use**							
No	651 (55.7)	101 (15.5)	Referent		309 (47.5)	Referent	
Yes	471 (40.3)	82 (17.4)	1.15 [0.83, 1.59]	0.390	213 (45.2)	0.90 [0.70, 1.16]	0.434
**Marijuana Use Weekly**							
No	807 (69.0)	124 (15.4)	Referent		365 (45.2)	Referent	
Yes	362 (31.0)	68 (18.8)	1.23 [0.88, 1.70]	0.225	177 (48.9)	1.09 [0.84, 1.42]	0.506
**AUDIT Score**							
Mean (SD)	6.84 (7.76)	7.65 (9.00)	1.02 [1.00, 1.04]	0.104	6.56 (7.19)		
Median [Min, Max]	4.00 [0, 40.0]	4.00 [0, 36.0]			4.00 [0, 35.0]	0.99 [0.97, 1.01]	0.227
**Depression Scale (CES-D)**							
Mean (SD)	16.4 (11.0)	18.4 (11.8)	1.02 [1.01, 1.03]	0.004	15.0 (10.8)	0.98 [0.97, 0.99]	<0.001
Median [Min, Max]	14.0 [0, 59.0]	16.0 [0, 59.0]			12.0 [0, 56.0]		
**Experienced Homophobia Scale**							
Mean (SD)	53.2 (31.5)	51.0 (32.4)	1.00 [0.99, 1.00]	0.164	55.1 (30.4)	1.00 [1.00, 1.01]	0.194
**Experienced Racism Scale**							
Mean (SD)	49.5 (24.0)	46.0 (25.0)	0.99 [0.98, 1.00]	0.010	51.5 (23.8)	1.01 [1.00, 1.01]	0.0418
**Internalized Homophobia Scale**							
Mean (SD)	15.6 (7.01)	16.4 (7.49)	1.02 [1.00, 1.05]	0.053	14.8 (6.75)	0.97 [0.96, 0.99]	0.002

* OR = Odds ratio.

**Table 2:** Associations between Recent Incarceration and Types of Networks at 12 Months Follow Up (N = 1169).

Network Type	% with Network Type	RR * (95% CI)	aRR ** (95% CI)	
**Small Medical Support Network**				<0.001 (0.035)
No Recent Incarceration	501 (49.9)	Referent	Referent	
Recent Incarceration	106 (64.2)	1.26 [1.12, 1.41]	1.16 [1.01, 1.34]	
**Small Financial Support Network**				<0.001 (0.013)
No Recent Incarceration	481 (47.9)	Referent	Referent	
Recent Incarceration	104 (63.0)	1.28 [1.14, 1.45]	1.18 [1.04, 1.35]	
**Small Social Support Network**				0.151 (0.326)
No Recent Incarceration	256 (25.5)	Referent	Referent	
Recent Incarceration	51 (30.9)	1.19 [0.94, 1.52]	1.15 [0.87, 1.52]	
**Small Emotional Support Network**				0.087 (0.916)
No Recent Incarceration	379 (37.7)	Referent	Referent	
Recent Incarceration	74 (44.8)	1.17 [0.98, 1.40]	1.01 [0.81, 1.26]	
**Consistent Network Support**				0.010 (0.337)
No Recent Incarceration	483 (48.1)	Referent	Referent	
Recent Incarceration	59 (35.8)	0.77 [0.63, 0.94]	0.90 [0.73, 1.12]	
**At Least One Person in Network Providing Each Form of Support**				0.033 (0.582)
No Recent Incarceration	666 (66.3)	Referent	Referent	
Recent Incarceration	94 (57.0)	0.87 [0.77, 0.99]	0.97 [0.86, 1.09]	
**One Person or Fewer in Network Providing Each Form of Support**				0.094 (0.271)
No Recent Incarceration	157 (15.6%)	Referent	Referent	
Recent Incarceration	35 (21.2%)	1.32 [0.95, 1.81]	1.24 [0.85, 1.81]	

* RR = Risk ratio. ** aRR = Adjusted Risk Ratio; Models adjusted for covariates using IPTW: included study site; age; gender identity; unstable housing; education; insufficient income; hard drug use in the past six months; weekly marijuana use; current health coverage; lifetime incarceration; AUDIT score; Depression scale score; physical and/or threatened violence due to race and/or sexuality; perceived racism and homophobia; internalized homophobia; social support scale score; sex with female partners in the past six months; having received HIV testing; transactional sex in the past six months; multiple partnerships; concurrent partnership; currently cohabiting with primary partner. Biologically ascertained covariates included HIV status at baseline and any STI (i.e., syphilis assessed via blood testing, and gonorrhea and chlamydia assessed via urine/rectal swab testing).

**Table 3:** Associations between Types of Networks and Health Outcomes at 12 Months Follow Up (N = 1169).

Network Type	% with Health Outcome	PR * (95% CI)	aPR (95% CI) **	Unadjusted (Adjusted) *p*-Value
**Multiple Sexual Partnerships**				
**Small Medical Support Network** No Yes	191 (43.8) 261 (43.0)	Referent 1.00 [0.87, 1.15]	Referent 0.92 [0.80, 1.05]	0.978 (0.227)
**Small Financial Support Network** No Yes	197 (43.0) 255 (43.6)	Referent 1.02 [0.89, 1.17]	Referent 0.94 [0.82, 1.07]	0.740 (0.340)
**Small Social Support Network** No Yes	342 (43.6) 110 (35.8)	Referent 0.77 [0.65, 0.91]	Referent 0.77 [0.65, 0.90]	0.002 (0.001)
**Small Emotional Support Network** No Yes	249 (42.2) 203 (44.8)	Referent 1.03 [0.97, 1.09]	Referent 0.98 [0.86, 1.12]	0.403 (0.792)
**Consistent Network Support** No Yes	243 (48.5) 209 (38.6)	Referent 0.90 [0.85, 0.96]	Referent 0.83 [0.72, 0.95]	<0.001 (0.008)
**At Least One Person in Network Providing Each Form of Support** No Yes	132 (46.6) 320 (42.1)	Referent 0.95 [0.89, 1.02]	Referent 0.97 [0.84, 1.13]	0.135 (0.707)
**One Person or Fewer in Network Providing Each Form of Support** No Yes	378 (44.4) 74 (38.5)	Referent 0.94 [0.87, 1.02]	Referent 0.80 [0.67, 0.96]	0.135 (0.018)
**Hard Drug Use**				
**Small Medical Support Network** No Yes	124 (28.4) 203 (33.4)	Referent 1.19 [1.00, 1.42]	Referent 1.05 [0.89, 1.24]	0.054 (0.541)
**Small Financial Support Network** No Yes	131 (28.6) 196 (33.5)	Referent 1.17 [0.98, 1.40]	Referent 1.07 [0.91, 1.26]	0.079 (0.388)
**Small Social Support Network** No Yes	227 (30.8) 100 (32.6)	Referent 1.07 [0.89, 1.29]	Referent 0.95 [0.80, 1.12]	0.465 (0.511)
**Small Emotional Support Network** No Yes	173 (29.3) 154 (34.0)	Referent 1.05 [0.99, 1.12]	Referent 1.03 [0.88, 1.20]	0.083 (0.717)
**Consistent Network Support** No Yes	174 (34.7) 153 (28.2)	Referent 0.93 [0.88, 0.99]	Referent 0.87 [0.74, 1.02]	0.021 (0.082)
**At Least One Person in Network Providing Each Form of Support** No Yes	95 (33.6) 232 (30.5)	Referent 0.97 [0.91, 1.03]	Referent 0.89 [0.75, 1.06]	0.340 (0.181)
**One Person or Fewer in Network Providing Each Form of Support** No Yes	269 (31.6) 58 (30.2)	Referent 0.99 [0.92, 1.07]	Referent 0.92 [0.75, 1.12]	0.837 (0.403)
**Unhealthy Alcohol Use**				
**Small Medical Support Network** No Yes	87 (20.0) 152 (25.0)	Referent 1.25 [0.99, 1.56]	Referent 1.20 [0.96, 1.51]	0.057 (0.108)
**Small Financial Support Network** No Yes	100 (21.8) 139 (23.8)	Referent 1.13 [0.91, 1.41]	Referent 1.15 [0.94, 1.41]	0.266 (0.175)
**Small Social Support Network** No Yes	169 (23.0) 70 (22.8)	Referent 1.00 [0.79, 1.26]	Referent 1.02 [0.82, 1.26]	0.974 (0.892)
**Small Emotional Support Network** No Yes	128 (21.7) 111 (24.5)	Referent 1.03 [0.98, 1.09]	Referent 1.16 [0.94, 1.42]	0.223 (0.156)
**Consistent Network Support** No Yes	125 (25.0) 114 (21.0)	Referent 0.96 [0.91, 1.01]	Referent 0.84 [0.69, 1.02]	0.086 (0.075)
**At Least One Person in Network Providing Each Form of Support** No Yes	72 (25.4) 167 (22.0)	Referent 0.96 [0.90, 1.02]	Referent 0.84 [0.67, 1.04]	0.198 (0.107)
**One Person or Fewer in Network Providing Each Form of Support** No Yes	191 (22.4) 48 (25.0)	Referent 1.03 [0.96, 1.10]	Referent 1.11 [0.86, 1.44]	0.406 (0.428)
**Visited ER in Past Six Months**				
**Small Medical Support Network** No Yes	82 (18.8) 125 (20.6)	Referent 1.10 [0.89, 1.37]	Referent 1.05 [0.84, 1.32]	0.376 (0.673)
**Small Financial Support Network** No Yes	81 (17.7) 126 (21.5)	Referent 1.19 [0.96, 1.47]	Referent 1.14 [0.92, 1.42]	0.110 (0.243)
**Small Social Support Network** No Yes	145 (19.7) 62 (20.2)	Referent 1.02 [0.81, 1.28]	Referent 0.95 [0.76, 1.20]	0.873 (0.680)
**Small Emotional Support Network** No Yes	105 (17.8) 102 (22.5)	Referent 1.08 [1.02, 1.15]	Referent 1.22 [0.99, 1.50]	0.015 (0.063)
**Consistent Network Support** No Yes	116 (23.2) 91 (16.8)	Referent 0.94 [0.88, 1.01]	Referent 0.89 [0.71, 1.11]	0.070 (0.305)
**At Least One Person in Network Providing Each Form of Support** No Yes	66 (23.3) 141 (18.6)	Referent 0.95 [0.89, 1.03]	Referent 0.88 [0.71, 1.10]	0.202 (0.271)
**One Person or Fewer in Network Providing Each Form of Support** No Yes	164 (19.3) 43 (22.4)	Referent 1.05 [0.97, 1.15]	Referent 1.10 [0.85, 1.42]	0.234 (0.457)
**Depression**				
**Small Medical Support Network** No Yes	133 (30.5) 212 (34.9)	Referent 1.16 [0.98, 1.37]	Referent 1.04 [0.88, 1.22]	0.086 (0.665)
**Small Financial Support Network** No Yes	132 (28.8) 213 (36.4)	Referent 1.06 [1.00, 1.13]	Referent 1.12 [0.96, 1.30]	0.006 (0.177)
**Small Social Support Network** No Yes	229 (31.1) 116 (37.8)	Referent 1.24 [1.05, 1.46]	Referent 1.16 [0.99, 1.36]	0.010 (0.071)
**Small Emotional Support Network** No Yes	184 (31.2) 161 (35.5)	Referent 1.27 [1.07, 1.51]	Referent 1.12 [0.95, 1.33]	0.066 (0.163)
**Consistent Network Support** No Yes	184 (36.7) 161 (29.7)	Referent 0.90 [0.85, 0.96]	Referent 0.85 [0.73, 0.99]	0.001 (0.035)
**At Least One Person in Network Providing Each Form of Support** No Yes	106 (37.5) 239 (31.4)	Referent 0.92 [0.86, 0.99]	Referent 0.90 [0.77, 1.06]	0.025 (0.221)
**One Person or Fewer in Network Providing Each Form of Support** No Yes	268 (31.5) 77 (40.1)	Referent 1.11 [1.02, 1.20]	Referent 1.22 [1.02, 1.46]	0.017 (0.031)

* PR = Prevalence ratio. ** aPR = Adjusted prevalence ratio; Models are adjusted for the following covariates measured at baseline: age, education, income, unstable housing, city of recruitment, reporting a place to go for medical care when sick, and having seen health care provider in the prior 6 months, and corresponding outcome variables reported at baseline.

## Data Availability

HPTN 061 data are available upon request: https://www.hptn.org/research/studies/hptn061/accesstostudydata.
